# Comparison of 2.0 mg/kg/day and 0.5 mg/kg/day immunosuppressive dexamethasone protocols as initial treatment for dogs with MUO

**DOI:** 10.3389/fvets.2025.1594310

**Published:** 2025-06-10

**Authors:** Miroslav Prikryl, Sara Ferrini, Petr Srenk

**Affiliations:** ^1^Department of Neurology and Neurosurgery, Vetino Jaggy Prague, Prague, Czechia; ^2^Sezione di Clinica Medica – Dipartimento di Scienze Veterinarie, Torino, Italy

**Keywords:** meningoencephalitis, MUO, dexamethasone, gastrointestinal, corticosteroids

## Abstract

**Introduction:**

Canine meningoencephalitis of unknown origin (MUO) is a common immune-mediated neurological disorder primarily treated with corticosteroids. However, the optimal initial dosing regimen remains unclear.

**Method:**

This prospective, randomized, parallel-group study evaluated the short-term clinical efficacy and gastrointestinal (GIT) safety of two intravenous dexamethasone dosing protocols (0.5 mg/kg/day vs. 2.0 mg/kg/day) in dogs diagnosed with MUO. Neurological and GI scoring systems were used to assess outcomes over a four-day hospitalization period.

**Results:**

Sixty dogs were enrolled and randomly assigned to either the 0.5 mg/kg/day (*n* = 30) or 2.0 mg/kg/day (*n* = 30) dexamethasone group. Neurological improvement was observed in 57 (95.0%) dogs, while 3 (5.0%) deteriorated, including 2 (3.3%) that died. No significant difference in neurological score changes was found between groups. Among the 58 survivors, 17 (28.3%) developed GIT signs, with 11 dogs in the 2.0 mg/kg/day group and 6 in the 0.5 mg/kg/day group. There was no significant difference in the incidence of GIT signs between groups, nor in the GIT score changes over time.

**Discussion:**

This study has not identified a significant difference in short term outcome using different dosing protocols of dexamethasone in dogs diagnosed with MUO. Further studies with larger sample sizes and extended follow-up periods are warranted to investigate the potential dose-dependent effects of dexamethasone on both neurological and GIT outcomes.

## 1 Introduction

Canine meningoencephalomyelitis of unknown origin (MUO) is a heterogeneous group of immune-mediated inflammatory diseases affecting the central nervous system (CNS) (1). Diagnosis relies on a combination of clinical signs, magnetic resonance imaging (MRI) findings, and cerebrospinal fluid (CSF) analysis, often supplemented by the exclusion of infectious etiologies (2, 3). Histopathologically, MUO is categorized into subtypes, most commonly granulomatous meningoencephalomyelitis (GME), necrotizing encephalitis (NE), or necrotizing leukoencephalitis (NLE) (4).

Treatment strategies primarily focus on immunosuppression, often using glucocorticoids such as dexamethasone or prednisolone (5). Both are frequently used intravenously (IV) at immunosuppressive doses to rapidly reduce inflammation and control clinical signs and followed by a tapering oral prednisolone regimen. The optimal tapering schedule and the selection of subsequent immunosuppressive agents (e.g., cytarabine, cyclosporine, azathioprine, mycophenolate mofetil, etc.) remain highly dependent on the individual patient’s response and practical considerations (5).

Dexamethasone is a highly potent glucocorticoid with virtually no mineralocorticoid activity and possesses some lipid antioxidant activity. The disposition of dexamethasone in dogs exhibits dose dependency. The elimination half-life is longer at a higher dose (0.1 mg/kg) compared with a lower dose (0.01 mg/kg), despite an increase in clearance; the differences presumably reflect the increase in the volume of distribution of dexamethasone at the higher dose (6). This could have potential clinical relevance concerning improvement in treatment as well as adverse effects. Common starting doses of dexamethasone for the treatment of immune-mediated diseases range from 0.1 to 1 mg/kg as a single dose or divided, with some authors reporting the use of up to 2 mg/kg/day during initial treatment (5). The rationale behind the high dose is based on the lympholytic properties of this dose, thus causing destruction of lymphocytes that otherwise would cause irreversible organ damage. The high dose is assumed to target the (nongenomic) metabolic processes necessary for sustained activity of lymphocytes, as opposed to the low (genomic) doses that target lymphocyte replication. Additionally, the high dose is considered to overcome glucocorticoid receptor saturation associated with chronic glucocorticoid therapy, causing significant glucocorticoid downregulation. Induction of T lymphocyte apoptosis may also occur (6), and all of these could be beneficial in patients treated for immune mediated disease.

Although dexamethasone is frequently used in veterinary and human medicine to treat immune-mediated diseases, data evaluating its efficacy and adverse effects in relation to MUO are lacking. There is also no evidence indicating whether the use of very high doses of dexamethasone, despite its pharmacological properties mentioned above, might provide better short-term or long-term outcomes in this subpopulation of patients. Therefore, this study aimed to assess the clinical efficacy of two dosing protocols during the hospitalization of patients with MUO using intravenous dexamethasone and to compare the gastrointestinal-related adverse effects of the treatment.

## 2 Materials and methods

We performed a randomized, open-label, parallel group-controlled trial.

### 2.1 Case selection

Dogs presented to the Vetino Jaggy Prague Clinic, Prague, Czech Republic, between May 2021 and January 2025 with a suspected diagnosis of MUO were actively recruited into the study. Data collected included signalment (age, breed, sex, and weight), duration of clinical signs before presentation, use of medications before referral, gastrointestinal signs observed, general physical, and neurological examination findings, and neuroanatomical localization. Onset of clinical signs was recorded as either peracute, acute, subacute, or chronic (24 h, 48 h, 7 days, and more than 7 days before presentation, respectively). Inclusion criteria were based on a clinical diagnosis of noninfectious meningoencephalomyelitis with a very high confidence level: dogs older than 6 months of age, small to toy breeds weighing less than 12 kg with neurological examination findings and neuroanatomical localization consistent with inflammatory brain or spinal cord disease (focal or multifocal predominantly asymmetric signs and localization), MRI results compatible with a non-infectious, inflammatory etiology (3, 7, 8), and CSF analysis with predominantly mononuclear or lymphocytic pleocytosis (more than 50% mononuclear cells) with a total nucleated cell count [TNCC] exceeding 15 cells/3 μL. Cell predominance was characterized as either mononuclear (more than 70% mononuclear cells) or mixed (50–70% mononuclear cells). Negative infectious agent testing was not mandatory; however, signalment, MRI and/or CSF evaluation had to lead to a high suspicion of a MUO diagnosis, otherwise, cases were excluded.

Additionally, dogs were excluded if they were older than 12 years, had received any immunosuppressants before presentation, tested positive for infectious causes before or after diagnosis (positive serological or PCR testing for Toxoplasma gondii, Neospora caninum, or positive CSF antibody titer for Tick-borne encephalitis virus), had predominantly neutrophilic pleocytosis in CSF, displayed signs of vomiting and diarrhea within 72 h before presentation, or did not stay hospitalized for at least 4 days for observation.

Dogs were still included if they had abnormal MRI findings compatible with inflammatory disease but normal CSF analysis, or if CSF sampling was not attempted because of concerns for increased intracranial pressure.

MRI was performed using a 0.2 Tesla scanner (Hitachi Airis 1). The imaging protocol included T2-weighted sagittal, dorsal, and transverse sequences; T1-weighted transverse sequences; T2-fluid-attenuated inversion recovery (FLAIR) transverse sequences; and post-contrast T1-weighted transverse images of the brain. For spinal cord imaging, T2-weighted sagittal and transverse sequences were obtained at a minimum.

Lesions were classified based on their location as follows: prosencephalon (including telencephalon and diencephalon), caudal fossa structures (comprising the mesencephalon, pons, myelencephalon, and cerebellum), and spinal cord. If lesions were present in two or more of these regions, they were categorized as multifocal.

All cases had serum biochemistry and cell blood count performed as a minimum database.

### 2.2 Interventions and randomization

Power analysis was not performed due to a lack of preliminary data. Therefore, a number of 30 dogs per group was set as an initial limit for analysis. Additional cases would then be collected in the event of marked tendency towards a significant difference between the groups.

Upon MUO diagnosis, written informed consent for study enrollment was obtained. As the dogs were enrolled, they received a number 1–60. Random Sequence Generator was used for randomization – each number (1–60) randomly assigned to one of the treatment strategies:

- Group L—dexamethasone at 0.5 mg/kg/day IV once daily for 4 days.
- Group H—dexamethasone at 2.0 mg/kg/day IV once daily for 4 days.

Both clinicians and owners were aware of the assigned treatment groups.

Both groups subsequently received a tapered PO prednisone course over 3 to 6 months starting at 2 mg/kg/day PO, according to the standard practice protocol.

### 2.3 Neurological and gastrointestinal scoring of dogs

A full neurological examination was performed and recorded at the time of admission by a neurology resident-in-training (MP) or by a board-certified neurologist (PS). A neurological scoring system was adapted from existing literature (9) and modified by the authors (Supplementary Table 1). Dogs were hospitalized for at least 4 days before discharge to allow for the assessment of neurological deficits and stool consistency. Neurological scoring was conducted daily and was performed by a board-certified neurologist (PS) and a neurology resident-in-training (MP), reaching an agreement (e.g., presence or absence of neurological deficit and its precise score based on scoring system). Assessments began 12 to 24 h after treatment initiation and continued until discharge. In the event of death, a total score of 14 (see Supplementary Table 1) was assigned to the dog for that day.

The GIT scoring system was adopted and adjusted from the Purina Fecal Scoring Chart (Supplementary Table 2). Scoring was based on stool consistency, evaluated either directly by the authors or through daily medical reports. These reports were compiled by trained nurses who documented stool characteristics, including consistency, color, and the possible presence of fresh or digested blood. Dogs were fed a commercial diet (Royal Canin Sensitivity Control Duck with Rice Loaf) during hospitalization. If dogs were known to be food intolerant, owners were asked to bring their regular home diet to the clinic.

### 2.4 Outcome measures

The primary outcome measure was the evaluation of neurological improvement or deterioration during hospitalization under two different dexamethasone dosing protocols.

The secondary outcome measure involved comparing GIT adverse effects between the two groups by assessing stool consistency using a scoring system. The evaluation was based on the most severe stool consistency observed each day.

### 2.5 Statistical analysis

#### 2.5.1 Baseline group comparisons

To assess the comparability of the two groups at baseline, we examined the distribution of demographic features and potential risk factors using statistical tests selected based on variable type and distribution. In particular, categorical variables (breed, sex, onset, neurological localization—both clinical and in MRI, CSF interpretation) were analyzed using the Chi-square test or Fisher’s exact test, as appropriate. On the other hand, continuous variables (age, weight, CSF TNCC), which did not meet the assumption of normality, were analyzed using the Wilcoxon rank-sum test.

These analyses ensured that the groups were statistically comparable prior to evaluating outcome measures (see Supplementary Table 4 for results).

#### 2.5.2 Outcome analysis

Here, our objective was to assess changes in clinical scores (neurological and gastrointestinal score) measured at four distinct time points, both within each group and between groups.

- Within-group changes over time were assessed using the Friedman test, a non-parametric alternative to repeated measures ANOVA. When significant, we performed post-hoc pairwise comparisons with adjusted *p*-values to control for multiple testing.
- Group × Time interaction effects were evaluated using the Aligned Rank Transform (ART) procedure. Unlike performing multiple Wilcoxon tests at each time point—an approach that fails to account for repeated measures—the ART method is specifically designed for factorial analysis of non-parametric data, analogous to a two-way repeated-measures ANOVA (10).

## 3 Results

### 3.1 Study sample

A total of 60 dogs were included in this study. The breeds represented were Yorkshire Terrier (28/60, 46.6%), Chihuahua (12/60, 20.0%), Maltese (5/60, 8.3%), Pomeranian (4/60, 6.7%), French Bulldog (4/60, 6.7%), Griffon Brabançon (2/60, 3.3%), Prague Rattle Dog (2/60, 3.3%), Poodle (1/60, 1.7%), Chinese Crested Dog (1/60, 1.7%), and Pug (1/60, 1.7%). The sex distribution was 34/60 (56.6%) females and 26/60 (43.4%) males, with 7/34 (20.6%) spayed females and 2/26 (7.7%) neutered males. The median age of the dogs at presentation was 4 years (IQR: 2–5 years). The median weight was 3.05 kg (IQR: 2.40–4.05 kg).

The onset of symptoms varied among the dogs, with chronic being the most common (21/60, 35.0%), followed by acute (18/60, 30.0%), subacute (18/60, 30.0%), and per-acute (3/60, 5.0%). Forty-nine out of 60 (81.7%) dogs had CSF analyzed, with 25/49 (51.0%) in Group H and 24/49 (49.0%) in Group L. Eleven out of 60 (18.3%) cases did not have CSF analyzed due to concerns of raised intracranial pressure or severe caudal fossa overcrowding. Median of TNCC/3 μL was 38 (IQR: 26–400 TNCC/3 μL). Of the analyzed cases, 77.6% (38/49) cases had mononuclear pleocytosis, 16.3% (8/49) had normal cell count, and 6.1% (3/49) had mixed pleocytosis.

Most common neuroanatomical localization was multifocal (36/60, 60.0%), followed by forebrain (17/60, 28.3%), brainstem (6/60, 10.0%) and T3-L3 spinal cord segments (1/60, 1.7%). Magnetic resonance imaging lesion localization was predominantly multifocal (35/60, 58.3%), followed by prosencephalon (21/60, 35.0%), caudal fossa structures (3/60, 5.0%), and spinal cord (1/60, 1.7%). Lesions within the cervical spinal cord were observed in 11/60 (18.3%) cases on MRI. Details on initial neurological examination findings can be seen in detail in Supplementary Table 3.

No significant differences were observed in demographics and clinical-diagnostic findings between Group L and Group H. Detailed group-wise data and statistical comparisons are available in Supplementary Table 4.

### 3.2 Treatment outcome

#### 3.2.1 Neuro-score

Over the four-day hospitalization period, 95% of the dogs (all but three) showed improvement in neurological scores. At day 1 (Time 0), the median neurological score was 7 (IQR: 5.75–7.25) for Group L and 7 (IQR: 5.75–8) for Group H. At day 2 (Time 1), the median score was 4 (IQR: 3–6.25) for Group L and 4 (IQR: 2.75–5.25) for Group H. At day 3 (Time 2), the median score was 3 (IQR: 1–4) for Group L and 2 (IQR: 1–4) for Group H. At day 4 (Time 3), the median score was 3 (IQR: 1–4) for Group L and 2 (IQR: 1–3) for Group H. In both groups, the score significantly decreased between Time 0 and Time 1 (Group L: *p* = 0.02; Group H: *p* = 0.02) and between Time 1 and Time 2 (Group L: *p* < 0.001; Group H: *p* = 0.01). The improvement between Time 2 and Time 3 was not significant.

**Figure 1:**
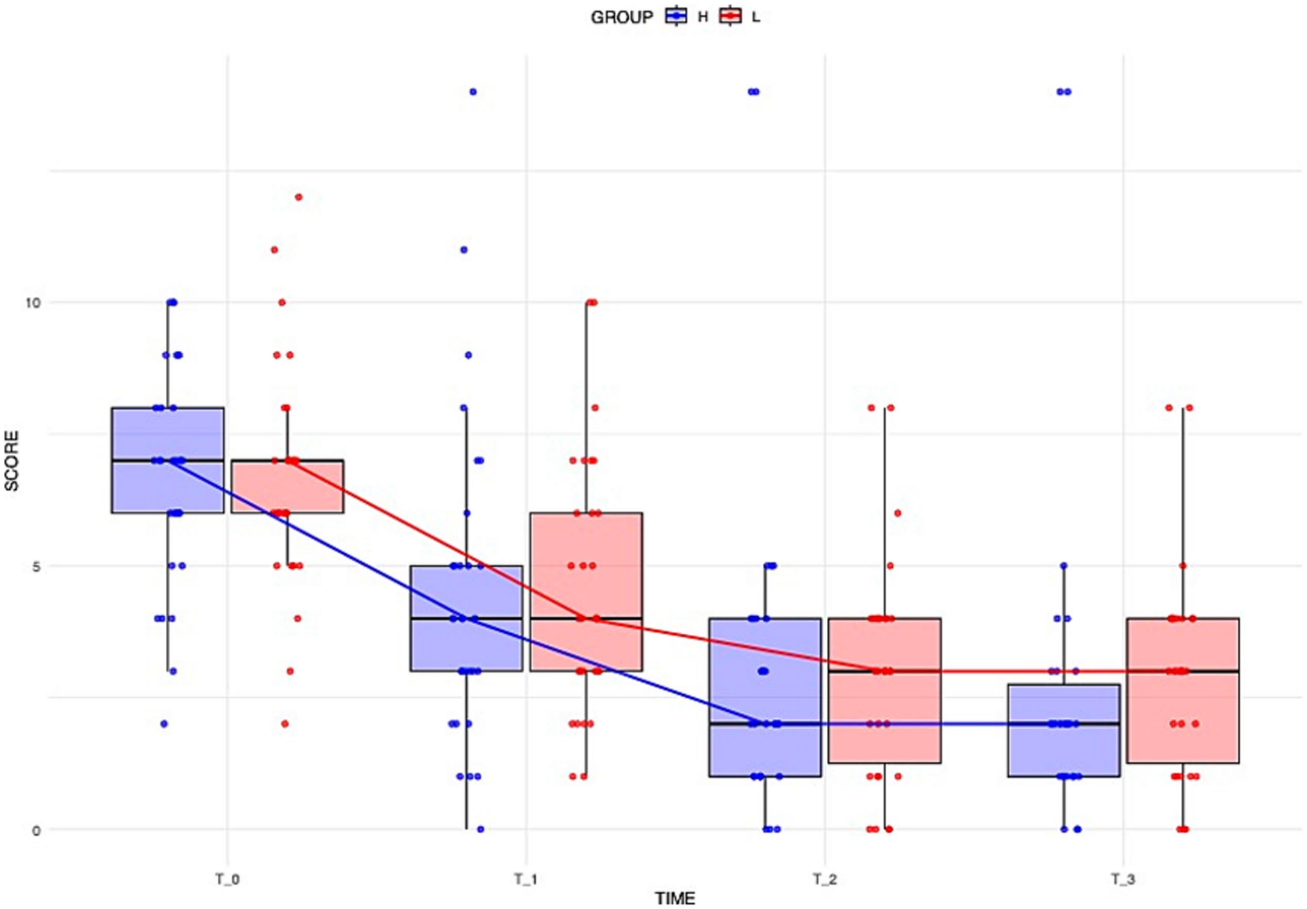
Distribution of dogs’ neurological scores over time across different groups. Boxplots illustrate the overall distribution of the score at each time point, with colors corresponding to groups (blue = Group H, red = Group L). The box represents the IQR, while the black line inside each box indicates the median. Individual data points are displayed with jitter to avoid overlap, and the solid lines connect the median values at each time point for each group, providing a representation of the central trend over time.

Overall, the degree of improvement calculated through the ART model did not differ significantly between the Group L and the Group H (Figure 1). The calculated effect size for the treatment between the two groups was small (d = −0.186).

Three dogs either deteriorated (1/3, 33.3%) or died (2/3, 66.7%) during the observation period. The two dogs that died were in Group H — one died on day 2, and the other on day 3. One dog exhibited chronic brainstem dysfunction with progressive somnolence, with extensive MRI lesions of the brainstem, and severe CSF pleocytosis; it deteriorated neurologically and died due to respiratory arrest. The other presented with acute brainstem and cerebellar involvement, developed aspiration pneumonia, and did not survive despite aggressive supportive care. One dog deteriorated on day 4, initially presenting with a cluster of seizures, inconsistent menace responses and apathy, which progressed to absent menace response on both eyes and decreased postural reactions in all limbs. After the observation period, it received additional immunosuppressant (cyclosporin, 6 mg/kg, per os, BID) and improved.

Sixteen dogs had epileptic seizures as one of the main neurological symptoms. Out of those, 6 (37.5%) presented with epileptic seizures at admission, and received antiseizure medication during hospitalization period. Five dogs had cluster seizures and were treated with phenobarbital (Luminal, Desitin Arzneimitttel GmbH (DEU)) at an initial dose of 2–2.5 mg/kg twice daily. One dog presented with status epilepticus and received phenobarbital bolus (3 mg/kg IV) and a levetiracetam (Keppra 100 mg/mL, UCB Pharma S.A. (BEL)) bolus (30 mg/kg IV) as part of its initial stabilization before further diagnostics were performed. Following diagnostics, along with dexamethasone treatment, the dog continued receiving 2.5 mg/kg of phenobarbital IV twice daily. None of the 16 dogs experienced additional seizures after initiating treatment during hospitalization.

#### 3.2.2 GIT score

**Figure 2:**
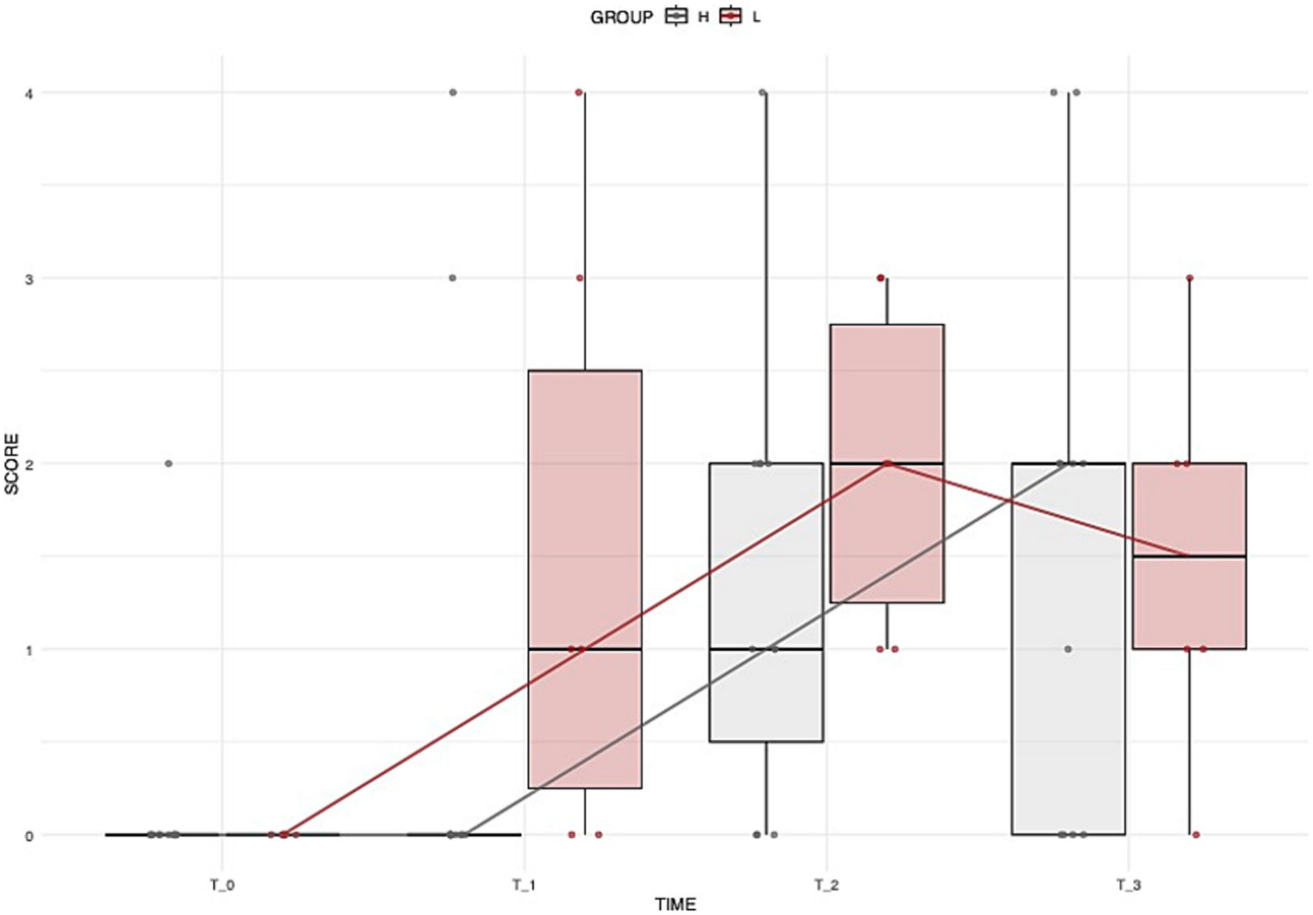
Distribution of dogs’ GIT scores over time across different groups. Boxplots illustrate the overall distribution of the score at each time point, with colors corresponding to groups (grey = Group H, brown = Group L). The box represents the IQR, while the black line inside each box indicates the median. Individual data points are displayed with jitter to avoid overlap, and the solid lines connect the median values at each time point for each group, providing a representation of the central trend over time.

The two dogs that died were excluded from the analysis of GIT signs. During the study, 17 of the 58 remaining dogs (31.0%) developed GIT signs, with 6/17 (35.3%) in Group L and 11/17 (64.7%) in Group H. No significant difference was found in the incidence of GIT signs between groups. Among dogs with GIT signs, the severity of symptoms did not differ significantly over time, either within or between treatment groups (Figure 2).

## 4 Discussion

This study evaluated the short-term effects of 2.0 mg/kg/day versus 0.5 mg/kg/day intravenous dexamethasone in 60 dogs diagnosed with MUO, over a 4-day hospitalization period. Neurological and GIT scoring systems were adapted from existing literature and used daily to assess efficacy and safety outcomes. While most dogs showed significant improvement over time, no statistically significant differences were observed between the groups.

The lack of a significant difference in neurological scores does not exclude the potential short-term efficacy of both protocols. Additionally, the small observed effect size related to treatment dosage suggests that higher dexamethasone doses may offer limited additional benefits in the short term.

Similarly, no statistically significant differences in GIT scores were observed between the treatment groups. However, a higher proportion of dogs in Group H (65%) experienced GIT side effects compared to Group L (35%). While this trend did not reach statistical significance, it is possible that GIT effects were partially influenced by dexamethasone dosage but that, due to the limited sample size, a statistically significant difference could not be detected. To ensure a more homogeneous study population and increase the likelihood of an MUO diagnosis, we selectively included small and toy breed dogs, excluding larger breeds. Larger breeds may be more severely affected by higher doses of dexamethasone, and the potential for increased side effects in these dogs remains unknown, warranting further investigation. Additionally, to reduce the risk of gastrointestinal disturbances during hospitalization, we implemented dietary management by providing a well-tolerated commercial diet. While no owners reported food intolerances in our study population, the possibility of undetected dietary sensitivities contributing to some cases of diarrhea cannot be ruled out.

A significant improvement in neurological scores was observed within the first 48 h of treatment in both groups. This finding may help guide owners in deciding whether and how long to hospitalize their dogs following a MUO diagnosis. Between days 3 and 4, the rate of improvement was not significant, and the median scores were already quite low (2 for Group H and 3 for Group L), suggesting that prolonged hospitalization beyond this period may not be necessary for all cases. However, since this study focused only on short-term outcomes, the precise timeline and extent of further neurological improvement remain unclear. Further studies comparing the effects of initial oral versus parenteral corticosteroid administration are needed to better understand the rate of neurological recovery in dogs with MUO.

There are no clear guidelines on initiation of treatment for patients diagnosed with MUO. Different dosing regimens are employed starting with anti-inflammatory doses of glucocorticosteroids while waiting for negative infectious agent test results, and then continuing with immunosuppression, or immediate immunosuppression using either prednisolone or dexamethasone right after obtaining the diagnosis. Initial treatment of immune-mediated conditions often starts with higher immunosuppression, commonly reported to be 4 mg/kg of prednisone or prednisolone per day for the first 4 to 7 days. As dexamethasone has 7 to 8 times greater glucocorticoid potency than prednisone (6), we decided to start with the upper immunosuppressive border of the dexamethasone dose in the Group L, reasoning that this would still predominantly target genomic metabolic processes. The Group H, receiving a very high immunosuppressive dose of 2.0 mg/kg/day, targeting also the nongenomic metabolic processes, would then be compared to the Group L, potentially resulting in different short-term outcome and adverse effects on the gastrointestinal tract (GIT). However, no significant differences were observed.

The neurological scoring system used in the present study was adapted from the neurodisability scale (NDS), recently developed as a clinical tool to guide clinicians and researchers to score patients with MUO (9). The NDS incorporates clinical signs frequently observed in MUO, including seizure activity, ambulatory status, posture, and deficits in cerebral, cerebellar, brainstem, and visual function. For the purpose of the present study, we found it useful to adjust the scoring system, as certain neurological deficits, such as postural reactions of the limbs, could result in some patients being classified as neurologically normal or underscored. To maintain consistency, all examinations were conducted by the first author (MP) and an ECVN diplomate (PS), ensuring agreement in scoring. Some dogs received adjuvant therapy that could have altered their scoring, such as antiseizure medication. Phenobarbital is known to cause transient lethargy and ataxia (11) and the authors were not blinded to the medications administered. However, to minimize bias, we initiated the treatment only if strictly necessary, and dogs were examined before receiving the medication to avoid evaluating them under immediate side effects.

Different glucocorticosteroid dosing protocols have also been studied in patients with multiple sclerosis (MS) (12, 13). As a condition analogous to MUO in dogs, MS is an autoimmune, inflammatory demyelinating disease of the central nervous system in humans, characterized in most cases by a relapsing–remitting course. The use of high-dose corticosteroids remains the cornerstone of treatment for MS relapses (14, 15). Various dosing regimens of methylprednisolone have been explored for MS relapse treatment (16–18) with most evidence supporting the efficacy of high-dose methylprednisolone, although there is still some inconsistency in defining what constitutes a “high dose.” Our study did not demonstrate a clear benefit of high-dose dexamethasone over the standard immunosuppressive dose during the hospitalization period. However, a larger sample size or longer treatment duration may yield different results. Additionally, unlike studies in MS patients, we did not assess the long-term outcomes of different initial dexamethasone protocols. Therefore, we cannot entirely rule out the potential advantage of one protocol over the other.

Short-term outcomes in dogs with MUO can be variable, with mortality rates reported to be as high as 33% within the first week. Previous studies have identified prognostic factors associated with poor short-term outcomes, including decreased mentation at presentation, the presence of seizures, and an increased percentage of neutrophils in CSF analysis. In our study, more than half of the dogs exhibited some degree of decreased mentation, and seizures were observed in a quarter of the population. Despite these risk factors, we observed a remarkably low mortality rate (3.3%) during the four-day observation period. However, our study exclusively included small and toy breed dogs, which may have contributed to the differences in outcomes compared to previous studies.

Moreover, young age and early diagnosis have been associated with better outcomes in MUO cases. Our findings support this, as the majority of our patients (65%) had clinical signs for less than 7 days and were relatively young (median age: 4 years), which may contribute to the higher short-term survival rate observed in our study.

Our study did not definitively exclude infectious causes. Some of our cases could have been affected by infectious agents, as the MRI findings and CSF pleocytosis type is non-specific to clearly differentiate infectious and non-infectious meningoencephalitis (19), and long-term outcome is not included in the analysis to further evaluate effect of immunosuppression. Despite this limitation, to minimize the risk, we applied strict inclusion criteria, selecting only dogs with a strong presumptive diagnosis of MUO. This approach aimed to improve diagnostic accuracy before initiating immunosuppressive treatment and to reduce the likelihood of misdiagnosis.

This study has some additional limitations. First, we selectively included only small and toy breed dogs, which may have influenced the results and limited their generalizability. However, since no clear advantage of one dosing protocol over the other is identified, the necessity of administering very high corticosteroid doses to large breed dogs remains uncertain, particularly given their potential for more severe side effects. Additionally, despite adequate training, stool consistency during nighttime hours was assessed by multiple technicians, introducing the possibility of variability in scoring. Although efforts were made to standardize assessments, inter-observer variability remains a potential limitation. Furthermore, data collection was limited to the four-day hospitalization period, meaning any delayed neurological deterioration or GIT side effects beyond this timeframe may have gone undetected. Lastly, authors were not blinded to the dose allocation of individual patients for practical reasons, and this could have resulted in performance and measurement bias, however we did not find significant differences between groups. Future studies should investigate long-term outcomes to provide a more comprehensive understanding of treatment efficacy and safety.

## 5 Conclusion

In conclusion, this study did not identify significant differences between 2.0 mg/kg/day and 0.5 mg/kg/day immunosuppressive doses of dexamethasone in terms of neurological improvement in patients with MUO. Regardless of the treatment group, 95% of dogs showed neurological improvement over the study period. Although no statistically significant differences were observed in GIT signs between groups, a higher percentage of dogs in the Group H developed gastrointestinal symptoms. Based on these findings, future studies with larger sample sizes and extended follow-up periods are warranted to further investigate the potential dose-dependent effects of dexamethasone on both neurological and GIT outcomes.

## Data Availability

The original contributions presented in the study are included in the article/Supplementary material, further inquiries can be directed to the corresponding author.

## Supplementary material

SUPPLEMENTARY TABLE 1 Neurological scoring system, adapted from Gonçalves et al. (2023).

SUPPLEMENTARY TABLE 2 Gastrointestinal (GIT) scoring system, adapted from Purina, Fecal Scoring Chart.

SUPPLEMENTARY TABLE 3 Detailed group-wise data on initial neurological examination findings based on the neurological scoring system.

SUPPLEMENTARY TABLE 4 Detailed group-wise demographic and clinical-diagnostic data with statistical comparison results.
