# Supplementary material for: Comparison of 2.0 mg/kg/day and 0.5 mg/kg/day immunosuppressive dexamethasone protocols as initial treatment for dogs with MUO
Source: Front Vet Sci. 2025 Jun 10;12:1594310. doi: 10.3389/fvets.2025.1594310 (PMC12185283; doi:10.3389/fvets.2025.1594310)
Supplement: SUPPLEMENTARY TABLE 1 — Neurological scoring system, adapted from Gonçalves et al. (2023). [file Table_1.docx]

| Neurological examination findings | | Score |
| --- | --- | --- |
| Menace deficits | Unilateral | 1 |
|  | Bilateral | 2 |
| Seizures | Occasional | 1 |
|  | Clusters | 2 |
|  | Status epilepticus | 3 |
| Compulsive behaviour | Occasional | 1 |
|  | Constant | 2 |
| Pain | Mild | 1 |
|  | Severe | 2 |
| Postural reactions (overnuckling) | Unilateral Inconsistent | 1 |
|  | Unilateral Absent | 2 |
|  | Bilateral inconsistent | 2 |
|  | Bilateral absent | 3 |
| Mentation | Obtunded | 1 |
|  | Stuporous | 2 |
|  | Comatose | 3 |
| Nystagmus | Positional | 1 |
|  | Spontaneous | 2 |
| PLR deficits | Unilateral | 1 |
|  | Bilateral | 2 |
| Palpebral reflex | Reduced (unilateral or bilateral) | 1 |
|  | Absent (unilateral or bilateral) | 2 |
| Gag reflex | Reduced with dysphagia | 1 |
|  | Absent | 2 |
| Vestibular ataxia | Ambulatory | 1 |
|  | Ambulatory and falling | 2 |
|  | Non Ambulatory | 3 |
| Strabismus | Positional unilateral | 1 |
|  | Positional bilateral | 2 |
| Spinal cord | Ambulatory tetra / paraparesis | 1 |
|  | Non Ambulatory tetra / paraparesis | 2 |
|  | Tetraplegia / paraplegia | 3 |

In case of death a total score of 14 (absent menace (2), bilateral absent postural reactions (3), comatose (3), absent PLR (2), absent palpebral reflex (2), absent gag reflex (2)).
