# Supplementary material for: Comparison of 2.0 mg/kg/day and 0.5 mg/kg/day immunosuppressive dexamethasone protocols as initial treatment for dogs with MUO
Source: Front Vet Sci. 2025 Jun 10;12:1594310. doi: 10.3389/fvets.2025.1594310 (PMC12185283; doi:10.3389/fvets.2025.1594310)
Supplement: SUPPLEMENTARY TABLE 2 — Gastrointestinal (GIT) scoring system, adapted from Purina, Fecal Scoring Chart. [file Table_2.docx]

| Score | Description | Look |
| --- | --- | --- |
| 0 | Firm, formed, almost no residue when picked up | 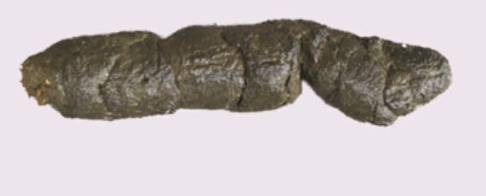 |
| 1 | Log shaped, moist surface, leaves residue on the ground | 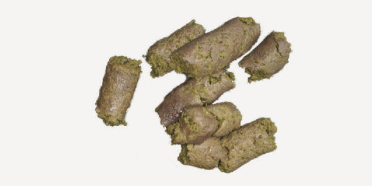 |
| 2 | Very moisty, remains distinct shape, looses form when picked up | 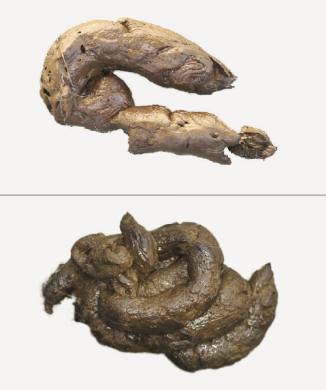 |
| 3 | Watery, no texture | 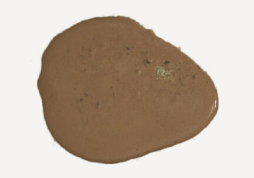 |
| 4 | Variably formed, presence of fresh or digested blood | 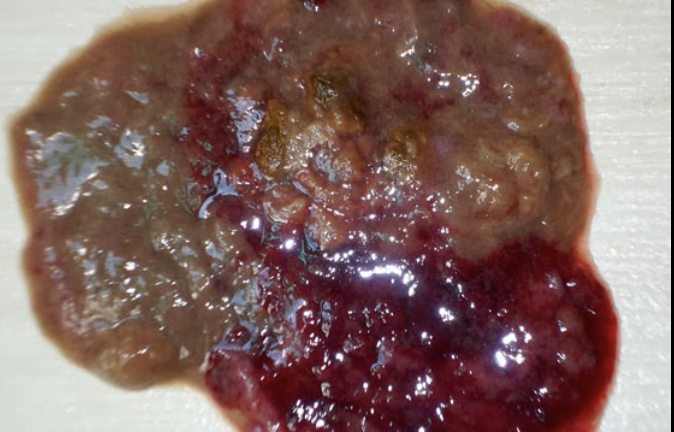 |
