# Supplementary material for: Comparison of 2.0 mg/kg/day and 0.5 mg/kg/day immunosuppressive dexamethasone protocols as initial treatment for dogs with MUO
Source: Front Vet Sci. 2025 Jun 10;12:1594310. doi: 10.3389/fvets.2025.1594310 (PMC12185283; doi:10.3389/fvets.2025.1594310)
Supplement: SUPPLEMENTARY TABLE 3 — Detailed group-wise data on initial neurological examination findings based on the neurological scoring system. [file Table_3.docx]

| Neurological examination findings | | Whole cohort (n=60) | Group L (n=30) | Group H (n=30) | Whole cohort (n=60) | Group L (n=30) | Group H (n=30) |
| --- | --- | --- | --- | --- | --- | --- | --- |
| Menace deficits | 1 Unilateral | 14 | 7 | 7 | 50 | 25 | 25 |
|  | 2 Bilateral | 36 | 18 | 18 |  |  |  |
| Seizures | 1 Occasional | 10 | 5 | 5 | 16 | 7 | 9 |
|  | 2 Cluster | 5 | 2 | 3 |  |  |  |
|  | 3 Status | 1 | 0 | 1 |  |  |  |
| Compulsive behaviour | 1 Occasional | 11 | 7 | 4 | 33 | 17 | 16 |
|  | 2 constant | 22 | 10 | 12 |  |  |  |
| Pain | 1 mild | 12 | 5 | 7 | 14 | 6 | 8 |
|  | 2 severe | 2 | 1 | 1 |  |  |  |
| Postural reactions (overnuckling) | 1 Unilateral Inconsistent | 12 | 5 | 6 | 50 | 24 | 26 |
|  | 2 Unilateral absent | 11 | 6 | 6 |  |  |  |
|  | 2 Bilateral inconsistent | 22 | 9 | 13 |  |  |  |
|  | 3 Bilateral absent | 6 | 4 | 1 |  |  |  |
| Mentation | 1 Obtunded | 27 | 15 | 12 | 32 | 18 | 14 |
|  | 2 Stuporous | 3 | 2 | 1 |  |  |  |
|  | 3 Comatose | 2 | 1 | 1 |  |  |  |
| Nystagmus | 1 positional | 5 | 2 | 3 | 6 | 2 | 4 |
|  | 2 spontaneous | 1 | 0 | 1 |  |  |  |
| PLR deficits | 1 unilateral | 3 | 1 | 2 | 7 | 4 | 3 |
|  | 2 bilateral | 4 | 3 | 1 |  |  |  |
| Palpebral reflex | 1 Reduced (unilateral or bilateral) | 5 | 4 | 1 | 6 | 5 | 1 |
|  | 2 Absent (unilateral or bilateral) | 1 | 1 | 0 |  |  |  |
| Gag reflex | 1 Reduced with dysphagia | 0 | 0 | 0 | 0 | 0 | 0 |
|  | 2 Absent | 0 | 0 | 0 |  |  |  |
| Vestibular ataxia | 1 ambulatory | 23 | 7 | 16 | 27 | 10 | 17 |
|  | 2 Ambulatory and falling | 2 | 1 | 1 |  |  |  |
|  | 3 Nonambulatory | 2 | 2 | 0 |  |  |  |
| Strabismus | 1 positional unilateral | 16 | 8 | 8 | 17 | 8 | 9 |
|  | 2 positional bilateral | 1 | 0 | 1 |  |  |  |
| Spinal cord | 1 Ambulatory tetra- / para paresis | 11 | 7 | 4 | 14 | 9 | 5 |
|  | 2 Nonambulatory tetra- / paraparesis | 3 | 2 | 1 |  |  |  |
