# Supplementary material for: Comparison of 2.0 mg/kg/day and 0.5 mg/kg/day immunosuppressive dexamethasone protocols as initial treatment for dogs with MUO
Source: Front Vet Sci. 2025 Jun 10;12:1594310. doi: 10.3389/fvets.2025.1594310 (PMC12185283; doi:10.3389/fvets.2025.1594310)
Supplement: SUPPLEMENTARY TABLE 4 — Detailed group-wise demographic and clinical-diagnostic data with statistical comparison results. [file Table_4.docx]

|  |  | **Group L (n = 30)** | **Group H (n = 30)** | **p value** |
| --- | --- | --- | --- | --- |
| Sex | Female | 18 (60,0%) | 16 (53,3%) | 0,88 |
|  | Female Spayed | 3 (10,0%) | 4 (13,3%) |  |
|  | Male | 12 (40,0%) | 14 (46,6%) |  |
|  | Male Neutered | 1 (3,3%) | 1 (3,3%) |  |
| Breed | Yorkshire Terrier | 17 (56,7%) | 11 (36,7%) | 0,28 |
|  | Chihuahua | 4 (13,3%) | 8 (26,7%) |  |
|  | Pomeranian | 3 (10,0%) | 1 (3,3%) |  |
|  | French Bulldog | 1 (3,4%) | 3 (10,0%) |  |
|  | Maltese | 1 (3,4%) | 4 (13,4%) |  |
|  | Pug | 1 (3,3%) | 0 (0%) |  |
|  | Chinese Crested Dog | 1 (3,3%) | 0 (0%) |  |
|  | Prague Rattle Dog | 1 (3,3%) | 1 (3,3%) |  |
|  | Poodle | 0 (0%) | 1 (3,3%) |  |
|  | Griffon Brabançon | 1 (3,3%) | 1 (3,3%) |  |
| Onset | Acute (A) | 12 (40,0%) | 6 (20,0%) | 0,39 |
|  | Chronic (CH) | 10 (33,3%) | 11 (36,7%) |  |
|  | Subacute (S) | 7 (23,4%) | 11 (36,7%) |  |
|  | Peracute (P) | 1 (3,3%) | 2 (6,6%) |  |
| MRI | Prosencephalon | 12 (40,0%) | 9 (30,0%) | 0,15 |
|  | Caudal fossa | 3 (10,0%) | 0 (0,0%) |  |
|  | Spinal cord | 0 (0,0%) | 1 (3,3%) |  |
|  | Multifocal | 15 (50,0%) | 20 (66,7%) |  |
| Neuroanatomical localisation | Brain stem | 3 (10,0%) | 3 (10,0%) | 0,93 |
|  | Forebrain | 8 (26,7%) | 9 (30,0%) |  |
|  | T3-L3 | 0 (0,0%) | 1 (3,3%) |  |
|  | Multifocal | 19 (63,3%) | 17 (56,7%) |  |
| CSF interpretation | Not analysed | 6 (20,0%) | 5 (16,7%) | 0,55 |
|  | Normal (TNCC less than 15/3µl) | 2 (6,7%) | 6 (20,0%) |  |
|  | Mononuclear pleocytosis | 20 (66,6%) | 18 (60,0%) |  |
|  | Mixed pleocytosis | 2 (6,7%) | 1 (3,3%) |  |
| Outcome | Improved | 29 (96,7%) | 28 (93,3%) | 0,49 |
|  | Worse / death | 1 (3,3%) | 2 (6,7%) |  |
| Age | Median (IQR) | 4 (3-5,75) | 3,5 (2-5) | 0,50 |
| Weight (kg)  CSF TNCC (cells/3µl) |  | 2,75 (2,2-3,875) | 3,15 (2,7-4,1) | 0,18 |
|  |  | 38,5 (27,75-79,5) | 37,0 (17-430) | 0,83 |
